# Specific gut microbiome signatures predict the risk of acute ischemic stroke

**DOI:** 10.3389/fnagi.2024.1451968

**Published:** 2024-11-08

**Authors:** Shicheng Yu, Jiayu Shi, Gaojie Yu, Jin Xu, Yiyao Dong, Yan Lin, Huijia Xie, Jiaming Liu, Jing Sun

**Affiliations:** ^1^Department of Geriatrics, The Second Affiliated Hospital and Yuying Children’s Hospital of Wenzhou Medical University, Wenzhou, Zhejiang, China; ^2^Department of Preventive Medicine, School of Public Health, Wenzhou Medical University, Wenzhou, Zhejiang, China

**Keywords:** acute ischemic stroke, biomarkers, gut microbiota, predict, transient ischemic attack

## Abstract

**Introduction:**

Numerous studies have reported alterations in the composition of gut microbiota in patients with acute ischemic stroke (AIS), with changes becoming more pronounced as the disease progresses. However, the association between the progression of transient ischemic attack (TIA) and AIS remains unclear. This study aims to elucidate the microbial differences among TIA, AIS, and healthy controls (HC) while exploring the associations between disease progression and gut microbiota.

**Methods:**

Fecal samples were collected from acute TIA patients (*n* = 28), AIS patients (*n* = 235), and healthy controls (*n* = 75) and analyzed using 16 s rRNA gene sequencing. We determined characteristic microbiota through linear discriminant analysis effect size and used the receiver operating characteristic (ROC) curve to assess their predictive value as diagnostic biomarkers.

**Results:**

Our results showed significant gut microbial differences among the TIA, AIS, and HC groups. Patients with AIS exhibited higher abundances of *Lactobacillus* and *Streptococcus,* along with lower abundances of *Butyricicoccaceae* and *Lachnospiraceae*_UCG-004. Further analysis revealed that the abundance of characteristic bacteria, such as *Lactobacillus* and *Streptococcus*, was negatively correlated with HDL levels, while *Lactobacillus* was positively correlated with risk factors such as homocysteine (Hcy). In contrast, the abundance of *Lachnospiraceae*_UCG-004 was negatively correlated with both Hcy and D-dimer levels. ROC models based on the characteristic bacteria *Streptococcus* and *Lactobacillus* effectively distinguished TIA from AIS, yielding areas under the curve of 0.699 and 0.626, respectively.

**Conclusion:**

We identified distinct changes in gut bacteria associated with the progression from TIA to AIS and highlighted specific characteristic bacteria as predictive biomarkers. Overall, our findings may promote the development of microbiome-oriented diagnostic methods for the early detection of AIS.

## 1 Introduction

Acute ischemic stroke (AIS) is a leading cause of death and long-term disability worldwide, driven by complex and multifaceted etiological factors, including metabolic disorders, alterations in gut microbiota, and systemic inflammation. Although these traditional risk factors are crucial, they do not fully account for the variability in AIS occurrence and outcomes. Notably, up to 15–30% of strokes are preceded by transient ischemic attack (TIA) (Rothwell et al., 2006; Rothwell and Warlow, 2005), which is characterized by a series of temporary neurological symptoms and serves as a strong predictor of subsequent stroke. The 90-day stroke risk after a TIA may be as high as 10–20%, with approximately half of these occurring within 2 days of the index event (Johnston et al., 2007; Hill et al., 2004). TIA, characterized by its suddenness, transience, and reversibility, is widely regarded as a high-risk factor for stroke and represents the optimal time window for prevention and intervention.

Due to the lack of precisely predictive biomarkers, a considerable proportion of TIA patients miss the critical window for preventive measures due to the lack of precise predictive biomarkers. Currently, the diagnosis of TIA primarily relies on medical history and imaging examinations. However, the insufficient sensitivity of imaging techniques and the potential for inaccurate recollection of symptoms by TIA patients create challenges for clinical diagnosis (Lima Filho et al., 2016; Amarenco et al., 2012). Therefore, there is an urgent need to explore novel biomarkers that can effectively screen high-risk TIA patients who may experience a stroke and evaluate disease progression from TIA to AIS.

Emerging evidence has begun to reveal the intricate relationship between altered gut microbiota and AIS, highlighting their significant potential to impact both AIS risk and recovery. Recent studies have shown that fecal microbiota transfer (FMT) from healthy microbiota can confer neuroprotective effects after a stroke (Singh et al., 2016; Lee et al., 2020). Additionally, it has been confirmed that stroke can induce dysbiosis in the gut microbiota and compromise epithelial barrier integrity (Peh et al., 2022; Pluta et al., 2021), leading to an exaggerated immune response that contributes to brain injury.

Furthermore, changes in gut microbiota have been widely associated with various brain diseases, especially in patients with stroke, where significant alterations in gut microbiota composition have been observed. Clinical studies have reported a notable decrease in the abundance of Bacteroidetes in individuals with AIS (Plovier et al., 2017). In addition, expansion of *Enterobacteriaceae* in the gut has been identified in both clinical and animal studies (Xu et al., 2021).

Furthermore, the dysbiosis of gut microbiota can, in turn, affect stroke outcomes, and the depletion of gut microbiota by antibiotic pretreatment exacerbates the prognosis of stroke (Winek et al., 2016; Honarpisheh et al., 2022). In addition, changes in the gut microbiota profile were associated with the diagnosis of AIS patients. Our previous study established a diagnostic model and identified potential microbial biomarkers for AIS patients with H-type hypertension (Yu et al., 2023). Moreover, we revealed the gut microbiota in patients with post-stroke depression (PSD) was characterized by increased genus *Streptococcus*, *Akkermansia,* and *Barnesiella*, which were diagnostic microbial biomarkers of PSD (Yao et al., 2023). Our previous study showed that the abundance of pro-inflammatory bacterial genera, such as *Streptococcus*, *Veillonella,* and *Acidaminococcus*, was increased in lacunar cerebral infarction patients (Ma et al., 2024). Emerging evidence has shown that the abnormal gut microbiota may be a cause or result of disease, suggesting that gut microbiota might provide biomarkers for detecting the risk or progression of AIS. However, previous studies merely focused on the link between the microbiota and TIA or AIS, which remains unexplored as a novel microbiome signature for early detection as a signal for the progression of TIA to AIS.

In this study, we aimed to characterize the alterations in gut microbiota among patients with TIA and AIS patients and further explored the associations between the characteristic microbiota and disease progression. These findings may encourage the development of microbiome-oriented diagnostics for predicting AIS.

## 2 Materials and methods

### 2.1 Participant recruitment

This study recruited 235 AIS patients, 28 TIA patients, and 75 healthy controls (HC) who had not experienced cerebrovascular events. The participants were recruited from the Second Hospital of Wenzhou Medical University in China from September 2020 to May 2024. The inclusion criteria were as follows: (1) admission within 72 h after AIS; (2) participants aged ≥18 years; (3) diagnosis of large-artery atherosclerosis confirmed by carotid artery ultrasound or transcranial Doppler sonography (TCD).

The exclusion criteria were as follows: (1) patients who had taken antibiotics or prebiotics that could disturb the gut microbiota within the past 3 months; (2) severe comorbid conditions (e.g., severe gastrointestinal disease, malignancy, respiratory failure, severe liver dysfunction, or renal failure), gastrointestinal surgery, or pregnancy; (3) clear causes of stroke or TIA unrelated to atherosclerosis (e.g., cervical artery dissection, cardiogenic cerebral infarction, perivascular procedural stroke, or other TOAST subtypes); (4) special dietary habits, such as vegetarianism.

AIS and TIA patients met the diagnostic criteria set by the American Heart Association/American Stroke Association (Sacco et al., 2013; Easton et al., 2009). All control participants underwent TCD, color Doppler flow imaging, and echocardiography to assess their cardiovascular health. The National Institutes of Health Stroke Scale (NIHSS) was used to assess the degree of neurological impairment (Zavaglia et al., 2015; Wu et al., 2015). This study was approved by the Medical Ethics Committee of the Second Affiliated Hospital of Wenzhou Medical University, and informed consent was obtained from all participants.

### 2.2 Demographic and clinical characteristics

The information on demographics (e.g., age and gender) was collected by trained researchers. Comorbidities like diabetes and hyperlipidemia (HL) were diagnosed by professionals of endocrinology. To determine lifestyle behaviors, we also collected participants’ histories of smoking or drinking. Moreover, laboratory indexes involved fasting glucose (FBG), glycosylated hemoglobin (HbAlc), homocysteine (Hcy), triglycerides (TG), high-density lipoprotein cholesterol (HDL), and D-dimer were measured and recorded.

### 2.3 Fecal collection and gut microbiota analysis

Each fecal sample (200 mg) was obtained from patients during inpatient or outpatient visits. Control group members provided stool samples voluntarily at the health screening center.

To ensure the quality of fecal samples, we used 2-mL sterile centrifuge tubes, froze them immediately in liquid nitrogen after collection, and stored them at −80°C. DNA was extracted from collected samples by the E.Z.N.A.^®^ Soil DNA Kit (Omega Bio-Tek, Norcross, GA, United States). For further detection, NanoDrop2000 (Thermo Scientific, Wilmington, United States) was used to determine the DNA concentration and purity. Then, the high mutation region of 16S ribosomal RNA (rRNA) was amplified. According to the manufacturer’s protocol, PCR products were recovered using 2% agarose gel, sequenced on the Illumina MiSeq platform (Illumina, San Diego, United States), and spliced using FLASH software.

Alpha diversity was quantified using the Ace and Shannon indices based on the Wilcoxon rank sum test. To further evaluate the overall ecology of the microbiome, *β*-diversity was calculated and visualized using a principal coordinates analysis (PCoA) diagram. The microbiota health index (MDI) was utilized to evaluate the degree of microbial dysbiosis, while microbiota composition was presented through bar charts at the phylum, family, and genus levels. In addition, linear discriminant analysis (LDA) effect size (LEfSe) was conducted to identify significant species capable of differentiating between groups, with a threshold LDA score set at >2. The correlation between the genus selected by LEfSe and clinical variables was visualized using a heatmap based on the Spearman correlation coefficient.

### 2.4 Statistical analysis

SPSS 26.0 software (SPSS, Chicago, United States) was used for the statistical analyses, and GraphPad Prism V.9.0.0 (La Jolla, CA, United States) was used for graph creation. PCoA diagram, MDI, and LDA analyses were carried out using R.^1^ Continuous variables follow a standard normal distribution (as inspected by the Kolmogorov–Smirnov test and the Shapiro–Wilk test) and were expressed by mean ± standard deviation (SD). As for the non-normally continuous variables, we used the non-parametric Mann–Whitney U-test or the Kruskal-Wallis test with Dunn’s correction for multiple comparisons to assess the significance of the differences among groups and expressed the result by the median and quartiles. Chi-square analysis assessed the significance of the differences between the groups for the nominal variables. Categorical variables were reported as the number of cases and percentages (%). A *p-*value of <0.05 was considered statistically significant.

## 3 Results

### 3.1 Study population characteristics at baseline

Eighteen participants were excluded due to the exclusion criteria, while nine individuals dropped out for failing to provide fecal samples or follow-up. The enrolled TIA patients had a median age of 65 years (Q57.5, 73.5) and were 50% female (14 out of 28). The demographic and laboratory data of the recruited participants are shown in Table 1.

**Table 1:** Demographic and clinical characteristics at baseline.

Variables HC (*n* = 75)		Stroke (*n* = 263)	
		TIA (*n* = 28)	AIS (*n* = 235)
Age (years)	64 (56.5, 72.0)	65 (57.5, 73.5)	68 (60.0, 76.0)
Male (%)	38 (50.7)	14 (50.0)	110 (46.8)
Diabetes (%)	15 (20.00)	6 (21.40)	72 (30.60)
Hypertension (%)	41 (54.70)	15 (53.60)	178 (75.70)
Hyperlipemia (%)	35 (46.70)	10 (35.70)	107 (45.50)
Smoking (%)	10 (13.30)	5 (17.90)	58 (24.70)
Drinking (%)	17 (22.70)	6 (21.40)	61 (26.00)
D-dimer (μg/mL)	0.35 (0.23, 0.54)	0.32 (0.24, 0.57)	0.38 (0.28, 0.61)
HbA1c (%)	5.72(5.40, 6.35)	5.72(5.38, 6.25)	5.80 (5.49, 6.80)
FBG (mmol/L)	4.75 (4.46, 5.77)	4.89 (4.40, 5.71)	5.16 (4.78, 6.32)
Hcy (μmol/L)	10.2 (8.4, 12.8)	10.3 (9.5, 13.9)	10.9 (8.8, 12.9)
TG (mmol/L)	1.38 (1.085, 2.08)	1.33 (1.15, 1.77)	1.48 (1.11, 1.96)
HDL (mol/L)	1.06 (0.87, 1.28)	1.09 (0.97, 1.33)	1.02 (0.88, 1.25)

HbA1c, glycosylated hemoglobin; FBG, fasting blood glucose; Hcy, homocysteine; TG, triglyceride; HDL, high-density lipoprotein.

### 3.2 Diversity and distribution of gut microbiota among three groups

**Figure 1:**
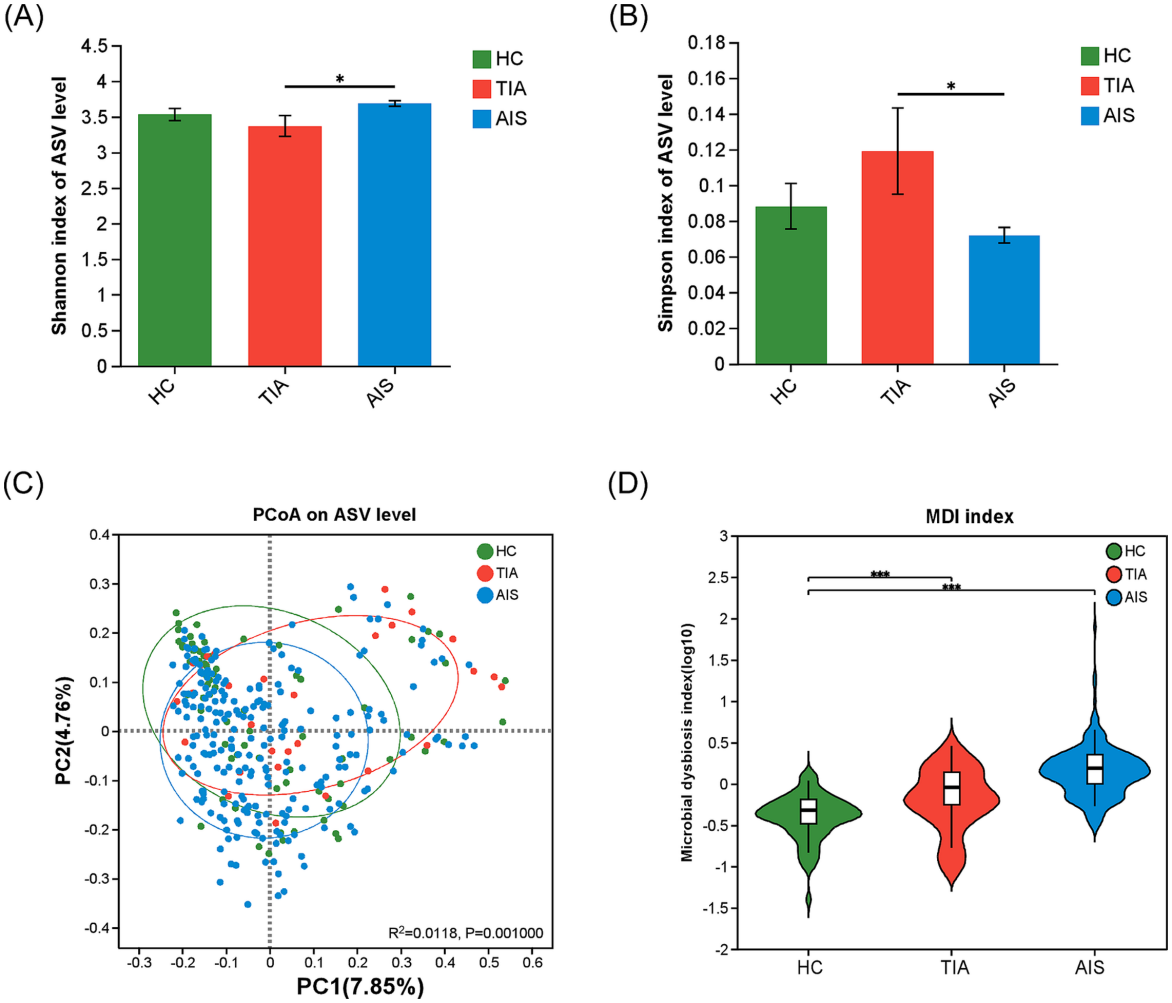
Microbial diversity analysis among HC, TIA, and AIS groups. (A,B) Alpha-diversity analysis among three groups, comparing Shannon and Simpson indices (Wilcoxon rank-sum test). (C) PCoA diagram on the ASV level analyzed the potential principal components affecting the differences in community composition. (D) Comparison of the Microbial dysbiosis index (MDI) among three groups. ^*^p < 0.05, ^***^p < 0.001.

The *α*-diversity indices (Shannon index and Simpson index) did not significantly differ between HC and TIA groups or between HC and AIS (*p* > 0.05) (Figures 1A,B). However, α-diversity between TIA and AIS groups showed significant differences that suggested the discrepancy in microbial composition (*p* < 0.05) (Figures 1A,B). The PCoA diagram on the ASV level analyzed the potential principal components affecting the differences in community composition and reflected an obvious separation trend in three groups (*p* < 0.05) (Figure 1C). The comparison of the MDI exhibited that the HC, TIA, and AIS groups had microbial dysbiosis (Figure 1D). In addition, MDI showed an increasing trend from TIA to AIS groups compared with HC.

**Figure 2:**
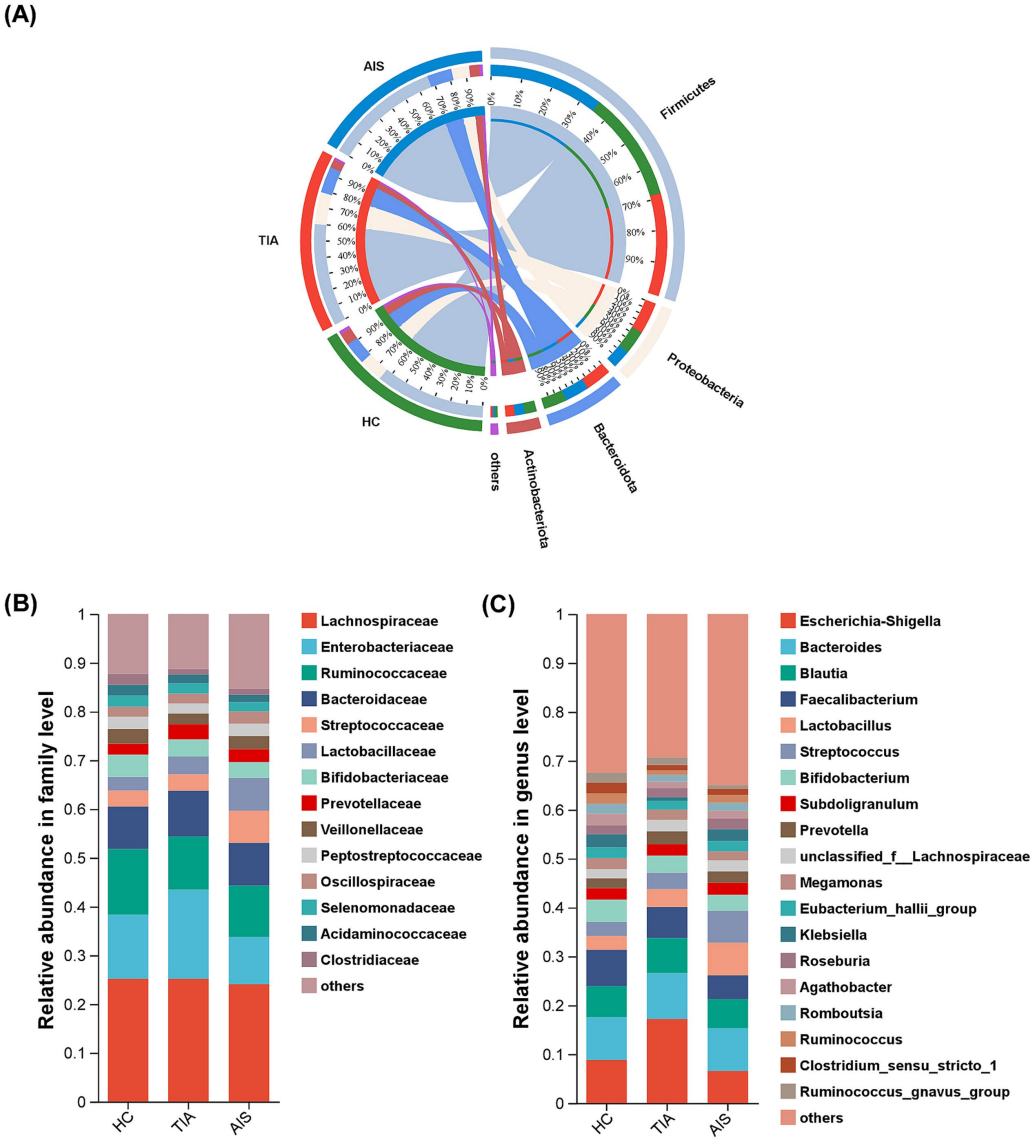
Microbial composition based on relative abundance among three groups. Circos diagram at the phylum level (A), bar diagram at the family level (B), and the genus level (C). Different colors and heights indicate the abundance ratios of different bacterial groups.

At the phylum level (Figure 2A), the microbiota detected in this study predominantly belong to four phyla: *Firmicutes*, *Proteobacteria*, *Bacteroidota*, and *Actinobacteriota*. Notably, *Proteobacteria* exhibited a higher abundance in the TIA group compared to the other groups. According to Figure 2B, at the family level, the taxa *Lachnospiraceae*, *Enterobacteriaceae*, *Ruminococcaceae*, *Bacteroidaceae*, *Streptococcaceae*, *Lactobacillaceae*, *Bifidobacteriaceae,* and *Prevotellaceae* collectively comprise approximately 70% of the total microbial composition across the three groups (Figure 2B). In the TIA group, *Enterobacteriaceae* showed a greater abundance relative to other groups, while *Ruminococcaceae* had a lower representation.

Moreover, the characterization at the genus level was more intricate; 19 genera, including *Escherichia-Shigella*, *Bacteroides*, *Blautia*, *Faecalibacterium*, *Lactobacillus*, *Streptococcus*, *Bifidobacterium*, and *Subdoligranulum*, accounted for 60% of the total bacterial population (Figure 2C). Notably, the proportion of *Escherichia-Shigella* in the TIA group was significantly increased.

### 3.3 The difference in characteristic bacteria among the three groups

**Figure 3:**
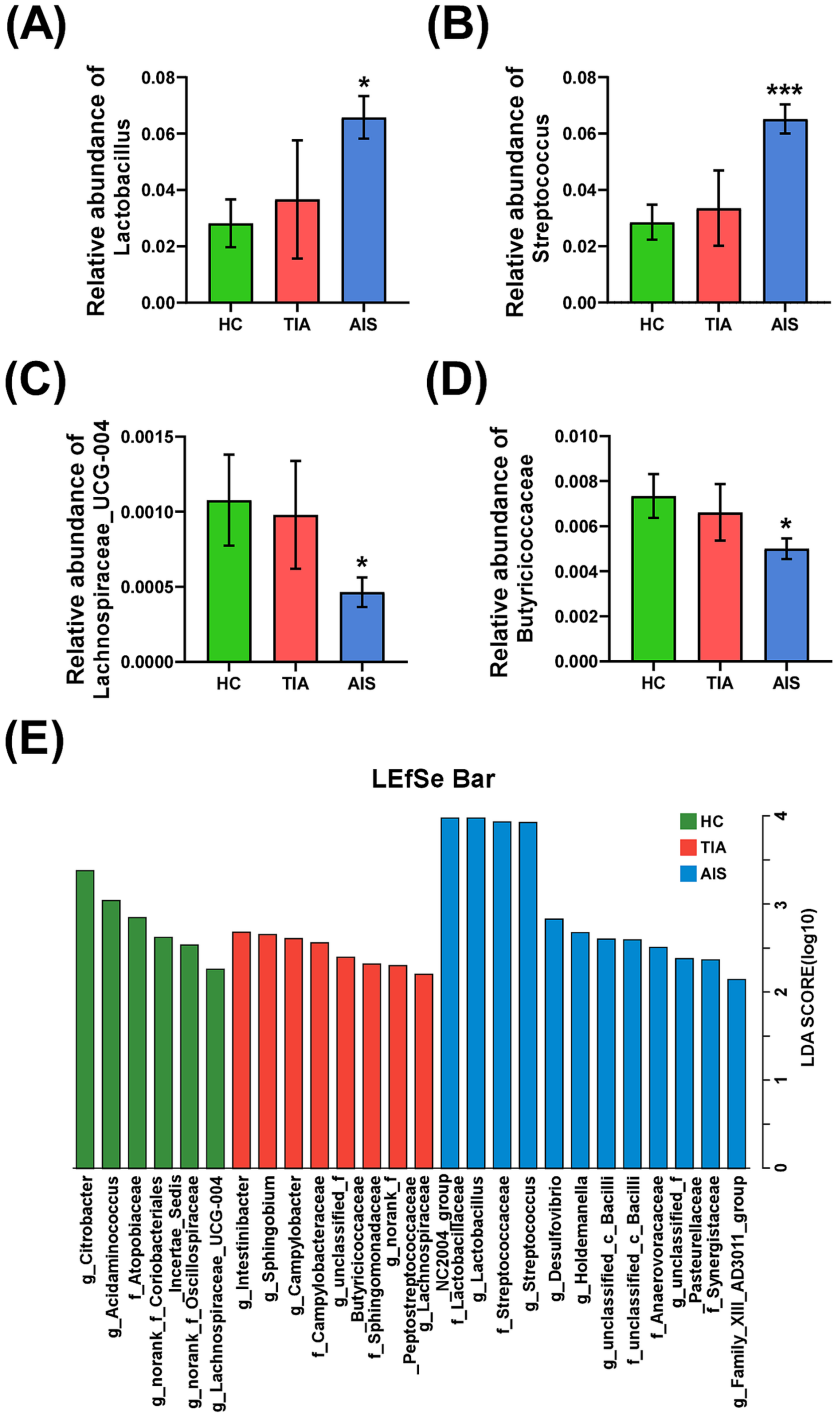
The difference of characteristic bacteria among the three groups. Histogram of bacterial differences based on Wilcoxon rank-sum test at family (D) and genus levels (A–C). (E) Histogram of LDA discrimination showing the phylogenetic relationships of bacteria taxa and LDA scores among three groups, based on LDA scores >2. ^*^p < 0.05, ^**^p < 0.01, ^***^p < 0.001.

The comparisons of differences among the three groups were exhibited at the genus level (Figures 3A–C) and the family level (Figure 3D). At the family level, *Butyricicoccaceae* (*p* < 0.05) displayed significant differences among the three groups (Figure 3D). At the genus level, *Lactobacillus* (*p* < 0.01), *Streptococcus* (*p* < 0.001), and *Lachnospiraceae_UCG-004* (*p* < 0.05) revealed significant differences (Figures 3A–C). In addition, *Lactobacillus* and *Streptococcus* showed a progressive increase tendency from HC and TIA to AIS, while *Lachnospiraceae_UCG-004* displayed a decrease tendency. LDA discriminant histogram analyzed multi-level differential species and used LDA value to measure the influence of species on the differential effect, suggesting that the species might play a key role in the occurrence and development of diseases. As shown in Figure 3E, the LDA discriminant histogram revealed the most significant taxa representing differences among the three groups from family to genus level. A total of 26 taxa with an LDA score of >2 were selected as characteristic bacteria to distinguish three groups (Figure 3E).

### 3.4 Correlation analysis between characteristic bacteria and clinical indicators

The heatmap showed that the abundance of *Lactobacillus* displayed an increasing trend across the HC, TIA, and AIS groups. It was significantly positively correlated with the stroke risk factor Hcy (*ρ* = 0.2235, *p* < 0.05) and negatively correlated with HDL (ρ = −0.2395, *p* < 0.05). Similarly, the abundance of *Streptococcus* exhibited a significant negative correlation with HDL (ρ = −0.1135, *p* < 0.05).

In contrast, the abundance of *Lachnospiraceae_UCG-004*, which exhibited a decreasing trend in the HC, TIA, and AIS groups, was significantly negatively correlated with the stroke risk factors D-dimer (ρ = −0.1393, *p* < 0.05) and Hcy (ρ = −0.1129, *p* < 0.05). Additionally, among the bacterial flora that increased in AIS patients compared to HC, the abundance of *unclassified_c__Bacilli* showed a significant positive correlation with D-dimer (ρ = 0.1125, *p* < 0.05), HbA1c (ρ = 0.1291, *p* < 0.05), and Hcy (ρ = 0.1243, *p* < 0.05).

**Figure 4:**
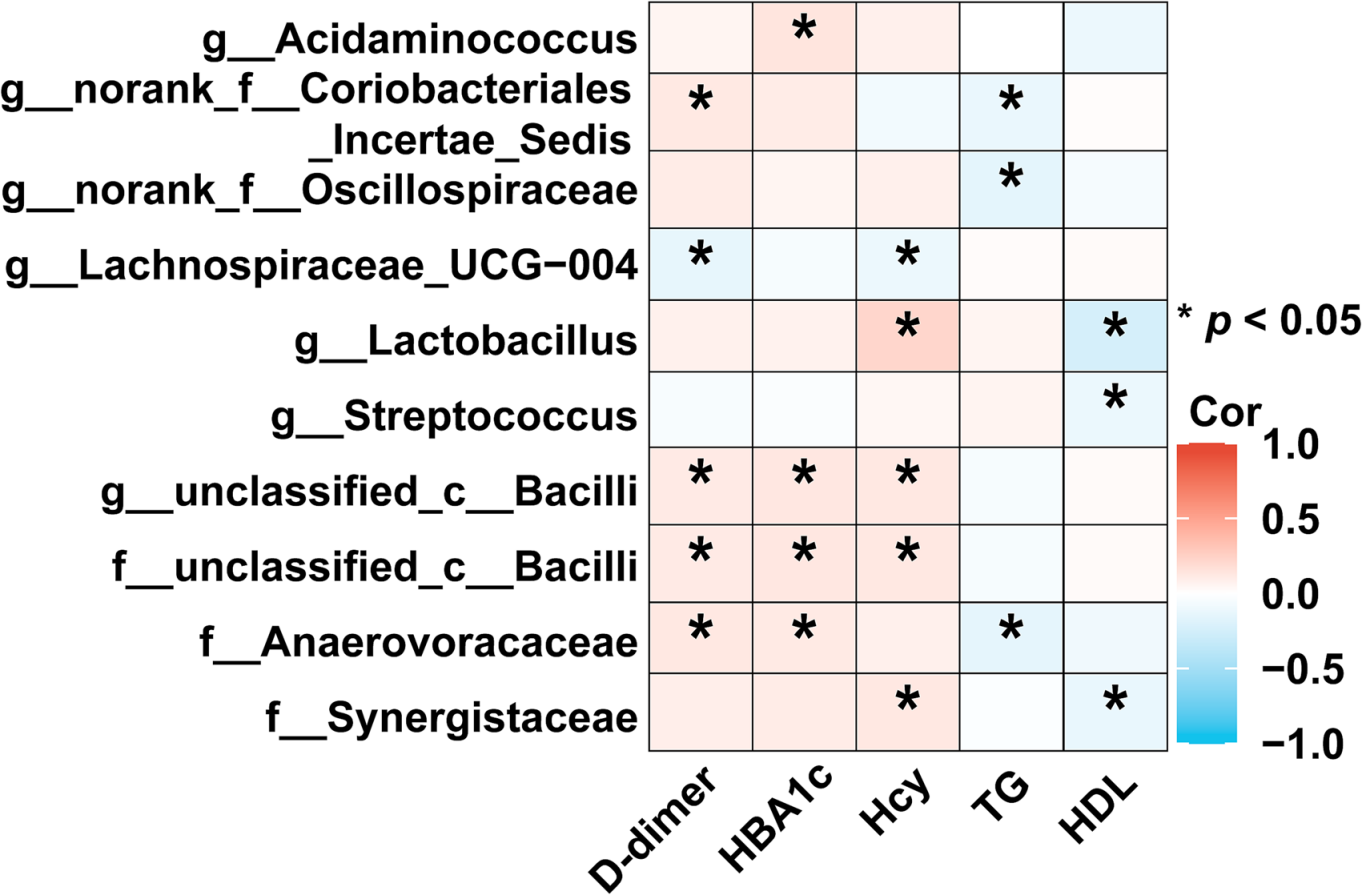
Correlation analysis between characteristic bacteria and clinical indicators. The heatmap revealed associations between differential bacteria and clinical factors (based on Spearman). The red color was positively correlated, and blue color was negatively correlated. Deeper colors indicated higher correlation values. ^*^p < 0.05, ^**^p < 0.01, ^***^p < 0.01.

The abundance of *Anaerovoracaceae* was also positively correlated with D-dimer (ρ = 0.1259, *p* < 0.05) and HbA1c (ρ = 0.1146, *p* < 0.05) while demonstrating a significant negative correlation with triglycerides (TG) (ρ = −0.1491, *p* < 0.05). Finally, the abundance of *Synergistaceae* exhibited a significant negative correlation with HDL (ρ = −0.1253, *p* < 0.05) (Figure 4).

### 3.5 Microbial biomarkers for prediction of AIS patients

**Figure 5:**
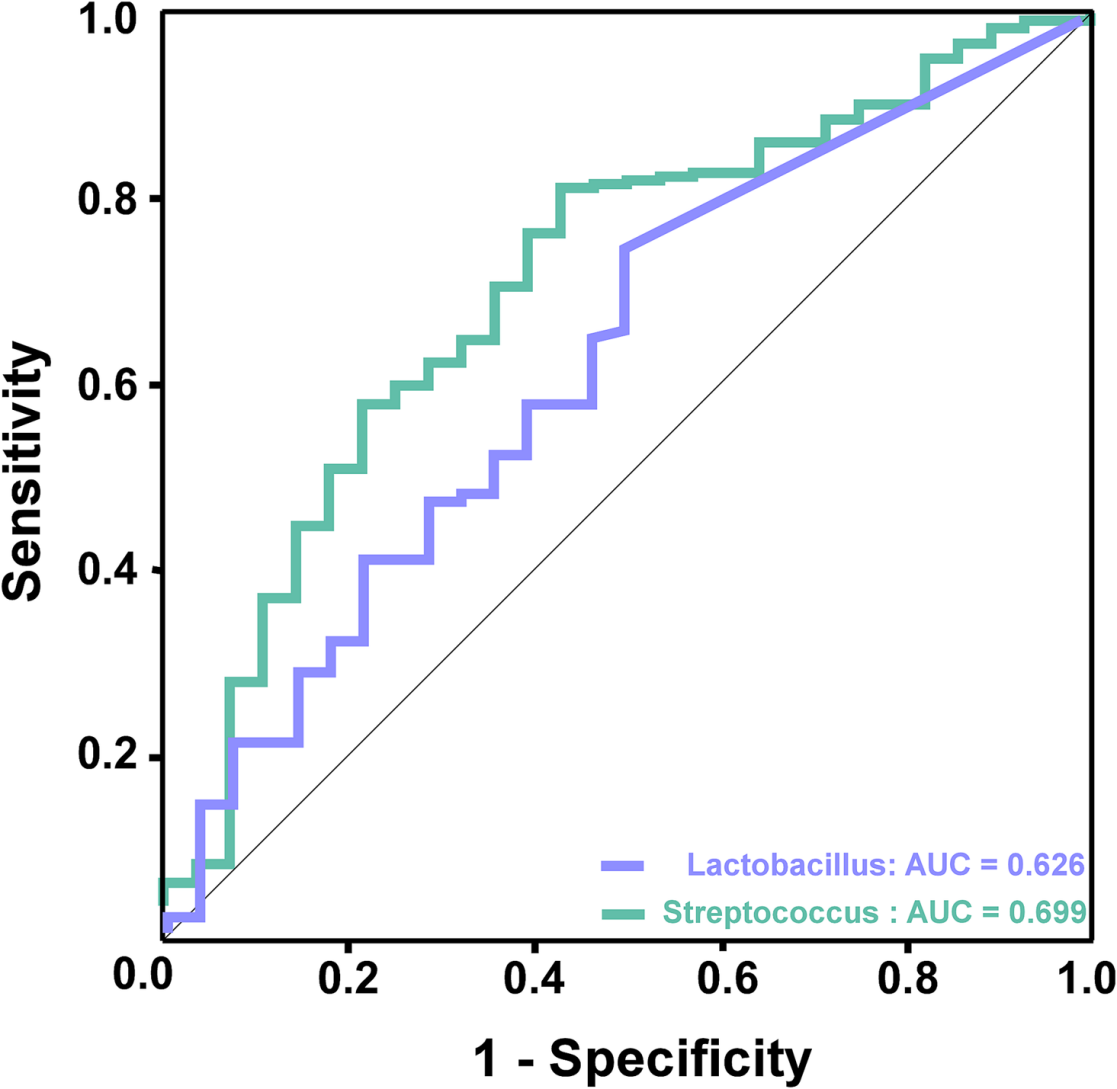
ROC curve analysis of potential microbial biomarkers for prediction of AIS patients. The green line represented *Streptococcus*, which achieved an AUC value of 0.699 (*p* < 0.05), and the purple line represented *Lactobacillus*, which achieved an AUC value of 0.626 (*p* < 0.05).

According to the LDA discriminant histogram and heatmap diagram, we screened out *Lactobacillus* and *Streptococcus* as the significant genus to distinguish TIA from AIS. As shown in Figure 5, *Lactobacillus* achieved an AUC value of 0.626 (*p* < 0.05), and *Streptococcus* achieved an AUC value of 0.699 (*p* < 0.05).

## 4 Discussion

In this study, gut microbial diversity in the TIA and AIS groups exhibited significant distinctions compared with the HC group.

AIS patients exhibited an enriched abundance of *Lactobacillus* and *Streptococcus,* along with a decreased abundance of *Lachnospiraceae*_UCG-004. Moreover, further study revealed characteristic bacteria: the abundance of Lactobacillus and Streptococcus was negatively correlated with HDL, and *Lactobacillus* was positively correlated with risk factors Hcy. In contrast, the abundance of *Lachnospiraceae*_UCG-004 negatively correlated with Hcy and D-dimer. Notably, ROC models based on the characteristic bacteria *Lactobacillus* and *Streptococcus* could effectively distinguish AIS patients from TIA patients. These results indicated that gut bacteria had the potential clinical utility to identify TIA patients who were likely to develop AIS.

Accumulating evidence indicated that gut microbiota played important roles in the occurrence and development of AIS. Our previous studies have demonstrated that gut microbiota composition significantly influences the outcome of AIS (Yu et al., 2023; Yao et al., 2023; Ma et al., 2024; Shi et al., 2023). Furthermore, the microbial composition significantly changes during AIS (Xu et al., 2021). Microbial alterations were also observed in animals subjected to stroke, such as the middle cerebral artery occlusion (MCAO) model (Kim et al., 2020). This study showed that TIA and AIS patients, compared with healthy controls, exhibited distinct microbial diversity, an increased microbial dysbiosis index, and altered bacterial communities. Yin et al. revealed that patients with stroke and TIA showed significant dysbiosis of the gut microbiota (Yin et al., 2015). These findings suggested that altered gut microbiota might be implicated in the initiation and pathogenesis of stroke.

In this study, we characterized the dysbiosis of gut microbiota in the TIA and AIS groups, noting an enriched abundance of genera, such as *Streptococcus* and *Lactobacillus*, alongside a decreased abundance of short-chain fatty acids (SCFA)-producing bacteria, specifically *Butyricicoccaceae* and *Lachnospiraceae*_UCG-004, compared to the HC group. Notably, *Lactobacillus* and *Streptococcus* showed a progressive increase from HC to TIA and then to AIS, while *Butyricicoccaceae* and *Lachnospiraceae*_UCG-004 displayed a decreasing trend across the same groups.

*Streptococcus* is a Gram-positive bacterium (Good, 2020). Consistent with our findings, other studies reported an increased abundance of *Streptococcus* in stroke patients (Peh et al., 2022; Yu et al., 2023; Sun et al., 2023). It has been suggested that *Streptococcus* can lead to endocarditis, promoting the occurrence of ischemic stroke (Stöllberger et al., 2003; Cao and Bi, 2019). In addition, *Lactobacillus* also showed an upward trend across the three groups. *Lactobacillus* has been extensively studied for its beneficial effects on human health (Avall-Jääskeläinen and Palva, 2005; Slattery et al., 2019). However, the function of *Lactobacillus* can also vary depending on factors such as the host’s immune status, the specific strain of *Lactobacillus*, and the presence of underlying diseases (Goldstein et al., 2015). Consistent with our results, other studies have also indicated an increase in *Lactobacillus* among stroke patients (Yamashiro et al., 2017; Li et al., 2020; Ling et al., 2020).

Additionally, the abundance of *Lachnospiraceae*_UCG-004 in the AIS group decreased compared to the HC group. *Lachnospiraceae*_UCG-004 was involved in the production of SCFAs (Jin et al., 2019; Huang et al., 2020). It was reported that SCFAs could relieve symptoms of the diseases by reducing neurotoxicity and neuroinflammation (Alpino et al., 2024; Zhang et al., 2021).

The pathogenesis of AIS has not been fully understood, but inflammation plays a pivotal role in the occurrence and progression of stroke (Chaturvedi and De Marchis, 2024; Kelly et al., 2021; Candelario-Jalil et al., 2022). Intense neuroinflammation, which occurred during the acute phase of stroke, was associated with blood–brain barrier (BBB) breakdown, neuronal injury, and worse neurological outcomes (Simats and Liesz, 2022; Oh and Parikh, 2022). The systemic inflammatory state induced by metabolic disorders, such as higher Hcy, HBA1c, and TG, actively participates in AIS pathogenesis (Ge et al., 2022). *Streptococcus* was reported to trigger an inflammatory response by leading to systemic induction of interleukin (IL)-1 and profoundly exacerbated (50–90%) ischemic brain injury in rats and mice (Dénes et al., 2014). This is evidenced by the active secretion of a variety of inflammatory mediators by the adipose tissue in obese individuals, which compounds the effects of traditional AIS risk factors like hypertension and hyperlipidemia (Chait and den Hartigh, 2020). Emerging evidence indicated that dysbiosis of the gut microbiota could cause an imbalance of metabolites and thereby affect the progression of stroke. The abundance of *Lachnospiraceae*_UCG-004, an SCFA-producing bacteria, decreased in the AIS group compared with the HC group. SCFA reduction might have a detrimental role in the whole setting of systemic inflammation. Chen R et al. confirmed that supplementation with butyrate in model rats of AIS could effectively remodel the gut microbiota and intestinal permeability and improve neurological deficits (Chen et al., 2019). SCFAs could alleviate hypertension, mitigate systemic inflammation, and decrease aortic atherosclerotic lesion area, which revealed the important association between SCFA-producing bacteria and vascular diseases (Dalile et al., 2019; Hu et al., 2022; Frampton et al., 2020). Animals subjected to the MCAO model, which mimics ischemic stroke, have been reported to have lower levels of SCFAs (Deleu et al., 2021). SCFAs can decrease microglial activation and modulate the integrity of BBB (Duan et al., 2023). In this study, we elucidated potential increases in inflammatory bacteria and decreases in SCFA-producing bacteria during the progression from TIA to AIS. Consequently, abnormal gut microbiota may influence the occurrence and progression of stroke through inflammatory responses and metabolite production.

In this study, our results showed that several risk factors for AIS, such as elevated D-dimer, HBA1c, Hcy, and TG, were associated with specific bacterial characteristics. A recent study demonstrated that AIS risk factors, such as metabolic disorders, hypertension, diabetes, obesity, and systemic inflammation, have been related to gut microbiota dysbiosis (Battaglini et al., 2020). This dysbiosis subsequently exacerbates the outcomes of AIS. Additionally, we established a diagnostic model for AIS using several abundant bacteria, including *Lactobacillus* and *Streptococcus*; the AUC demonstrated satisfactory predictive performance. These findings suggest that AIS patients exhibit more significant differences in gut microbial composition compared to those with transient ischemic attack (TIA), indicating that characteristic bacteria may serve as diagnostic biomarkers for AIS.

However, this study still has several limitations. First, it was a single-center study, limiting the observation of dynamic changes between participants and their gut microbiota. Second, the correlation analysis between the microbiota and AIS did not establish a causal relationship. Further validation of the identified differential bacteria and metabolites is essential to elucidate the underlying mechanisms of their interactions. Finally, individual variations in dietary habits and drug use might influence the composition and function of gut microbiota. Therefore, further analyses are imperative to elucidate the nuanced relationship between gut microbiota and AIS. In addition, addressing these limitations through rigorous experimental design and larger-scale clinical studies will contribute to a more comprehensive understanding of the potential of characteristic bacteria in diagnosing AIS.

In conclusion, this study revealed that the gut microbiota of AIS and TIA patients undergo significant changes. Moreover, *Streptococcus* and *Lactobacillus* were microbial biomarkers for AIS, which was worthy of further study on clinical application. These findings assist in predicting AIS in TIA patients and facilitate early warnings for AIS.

## Data Availability

The datasets presented in this study can be found in online repositories. The names of the repository/repositories and accession number(s) can be found at: https://www.ncbi.nlm.nih.gov/, PRJNA1140893.

## Footnotes

^1^www.r-project.org

## References

[ref1] Alpino G. Pereira-Sol G. A. Dias M. M. E. Aguiar A. S. Peluzio M. (2024). Beneficial effects of butyrate on brain functions: a view of epigenetic. Crit. Rev. Food Sci. Nutr. 64, 3961–3970. doi: 10.1080/10408398.2022.2137776, PMID: 36287024

[ref2] Amarenco P. Labreuche J. Lavallée P. C. (2012). Patients with transient ischemic attack with ABCD2 <4 can have similar 90-day stroke risk as patients with transient ischemic attack with ABCD2 ≥4. Stroke 43, 863–865. doi: 10.1161/strokeaha.111.636506, PMID: 22156685

[ref3] Avall-Jääskeläinen S. Palva A. (2005). *Lactobacillus* surface layers and their applications. FEMS Microbiol. Rev. 29, 511–529. doi: 10.1016/j.fmrre.2005.04.003 15935509

[ref4] Battaglini D. Pimentel-Coelho P. M. Robba C. Dos Santos C. C. Cruz F. F. Pelosi P.. (2020). Gut microbiota in acute ischemic stroke: from pathophysiology to therapeutic implications. Front. Neurol. 11:598. doi: 10.3389/fneur.2020.00598, PMID: 32670191 PMC7330114

[ref5] Candelario-Jalil E. Dijkhuizen R. M. Magnus T. (2022). Neuroinflammation, stroke, blood-brain barrier dysfunction, and imaging modalities. Stroke 53, 1473–1486. doi: 10.1161/strokeaha.122.036946, PMID: 35387495 PMC9038693

[ref6] Cao G. F. Bi Q. (2019). Pediatric infective endocarditis and stroke: a 13-year single-center review. Pediatr. Neurol. 90, 56–60. doi: 10.1016/j.pediatrneurol.2018.07.001, PMID: 30420107

[ref7] Chait A. den Hartigh L. J. (2020). Adipose tissue distribution, inflammation and its metabolic consequences, including diabetes and cardiovascular disease. Front Cardiovasc Med 7:22. doi: 10.3389/fcvm.2020.00022, PMID: 32158768 PMC7052117

[ref8] Chaturvedi S. De Marchis G. M. (2024). Inflammatory biomarkers and stroke subtype: an important new frontier. Neurology 102:e208098. doi: 10.1212/wnl.0000000000208098, PMID: 38165352

[ref9] Chen R. Xu Y. Wu P. Zhou H. Lasanajak Y. Fang Y.. (2019). Transplantation of fecal microbiota rich in short chain fatty acids and butyric acid treat cerebral ischemic stroke by regulating gut microbiota. Pharmacol. Res. 148:104403. doi: 10.1016/j.phrs.2019.104403, PMID: 31425750

[ref10] Dalile B. Van Oudenhove L. Vervliet B. Verbeke K. (2019). The role of short-chain fatty acids in microbiota-gut-brain communication. Nat. Rev. Gastroenterol. Hepatol. 16, 461–478. doi: 10.1038/s41575-019-0157-3 31123355

[ref11] Deleu S. Machiels K. Raes J. Verbeke K. Vermeire S. (2021). Short chain fatty acids and its producing organisms: an overlooked therapy for IBD? EBioMedicine 66:103293. doi: 10.1016/j.ebiom.2021.103293, PMID: 33813134 PMC8047503

[ref12] Dénes Á. Pradillo J. M. Drake C. Sharp A. Warn P. Murray K. N.. (2014). *Streptococcus pneumoniae* worsens cerebral ischemia via interleukin 1 and platelet glycoprotein Ibα. Ann. Neurol. 75, 670–683. doi: 10.1002/ana.24146, PMID: 24644058

[ref13] Duan H. Wang L. Huangfu M. Li H. (2023). The impact of microbiota-derived short-chain fatty acids on macrophage activities in disease: mechanisms and therapeutic potentials. Biomed. Pharmacother. 165:115276. doi: 10.1016/j.biopha.2023.115276, PMID: 37542852

[ref14] Easton J. D. Saver J. L. Albers G. W. Alberts M. J. Chaturvedi S. Feldmann E.. (2009). Definition and evaluation of transient ischemic attack: a scientific statement for healthcare professionals from the American Heart Association/American Stroke Association stroke council; council on cardiovascular surgery and anesthesia; council on cardiovascular radiology and intervention; council on cardiovascular nursing; and the interdisciplinary council on peripheral vascular disease. The American Academy of Neurology affirms the value of this statement as an educational tool for neurologists. Stroke 40, 2276–2293. doi: 10.1161/strokeaha.108.192218 19423857

[ref15] Frampton J. Murphy K. G. Frost G. Chambers E. S. (2020). Short-chain fatty acids as potential regulators of skeletal muscle metabolism and function. Nat. Metab. 2, 840–848. doi: 10.1038/s42255-020-0188-7 32694821

[ref16] Ge Y. Zadeh M. Yang C. Candelario-Jalil E. Mohamadzadeh M. (2022). Ischemic stroke impacts the gut microbiome, ileal epithelial and immune homeostasis. iScience 25:105437. doi: 10.1016/j.isci.2022.105437 36388972 PMC9650036

[ref17] Goldstein E. J. Tyrrell K. L. Citron D. M. (2015). *Lactobacillus* species: taxonomic complexity and controversial susceptibilities. Clin. Infect. Dis. 60, S98–S107. doi: 10.1093/cid/civ072, PMID: 25922408

[ref18] Good M. F. (2020). *Streptococcus*: an organism causing diseases beyond neglect. PLoS Negl. Trop. Dis. 14:e0008095. doi: 10.1371/journal.pntd.0008095, PMID: 32437344 PMC7241690

[ref19] Hill M. D. Yiannakoulias N. Jeerakathil T. Tu J. V. Svenson L. W. Schopflocher D. P. (2004). The high risk of stroke immediately after transient ischemic attack: a population-based study. Neurology 62, 2015–2020. doi: 10.1212/01.wnl.0000129482.70315.2f 15184607

[ref20] Honarpisheh P. Bryan R. M. McCullough L. D. (2022). Aging microbiota-gut-brain Axis in stroke risk and outcome. Circ. Res. 130, 1112–1144. doi: 10.1161/circresaha.122.319983, PMID: 35420913 PMC9674376

[ref21] Hu T. Wu Q. Yao Q. Jiang K. Yu J. Tang Q. (2022). Short-chain fatty acid metabolism and multiple effects on cardiovascular diseases. Ageing Res. Rev. 81:101706. doi: 10.1016/j.arr.2022.101706 35932976

[ref22] Huang K. Yu W. Li S. Guan X. Liu J. Song H.. (2020). Effect of embryo-remaining oat rice on the lipid profile and intestinal microbiota in high-fat diet fed rats. Food Res. Int. 129:108816. doi: 10.1016/j.foodres.2019.108816, PMID: 32036900

[ref23] Jin M. Kalainy S. Baskota N. Chiang D. Deehan E. C. McDougall C.. (2019). Faecal microbiota from patients with cirrhosis has a low capacity to ferment non-digestible carbohydrates into short-chain fatty acids. Liver Int. 39, 1437–1447. doi: 10.1111/liv.14106, PMID: 30919578

[ref24] Johnston S. C. Rothwell P. M. Nguyen-Huynh M. N. Giles M. F. Elkins J. S. Bernstein A. L.. (2007). Validation and refinement of scores to predict very early stroke risk after transient ischaemic attack. Lancet 369, 283–292. doi: 10.1016/s0140-6736(07)60150-0, PMID: 17258668

[ref25] Kelly P. J. Lemmens R. Tsivgoulis G. (2021). Inflammation and stroke risk: a new target for prevention. Stroke 52, 2697–2706. doi: 10.1161/strokeaha.121.034388, PMID: 34162215

[ref26] Kim K. A. Kim D. Kim J. H. Shin Y. J. Kim E. S. Akram M.. (2020). Autophagy-mediated occludin degradation contributes to blood-brain barrier disruption during ischemia in bEnd.3 brain endothelial cells and rat ischemic stroke models. Fluids Barriers CNS 17:21. doi: 10.1186/s12987-020-00182-8 32169114 PMC7071658

[ref27] Lee J. d'Aigle J. Atadja L. Quaicoe V. Honarpisheh P. Ganesh B. P.. (2020). Gut microbiota-derived short-chain fatty acids promote poststroke recovery in aged mice. Circ. Res. 127, 453–465. doi: 10.1161/circresaha.119.316448, PMID: 32354259 PMC7415518

[ref28] Li H. Zhang X. Pan D. Liu Y. Yan X. Tang Y.. (2020). Dysbiosis characteristics of gut microbiota in cerebral infarction patients. Transl. Neurosci. 11, 124–133. doi: 10.1515/tnsci-2020-0117, PMID: 33312718 PMC7706127

[ref29] Lima Filho J. B. de Lima T. I. Luvizutto G. J. Pereira Braga G. Bazan R. (2016). ABCD2 score and secondary stroke prevention: meta-analysis and effect per 1,000 patients triaged. Neurology 86:697. doi: 10.1212/wnl.0000000000002411 26880812

[ref30] Ling Y. Gong T. Zhang J. Gu Q. Gao X. Weng X.. (2020). Gut microbiome signatures are biomarkers for cognitive impairment in patients with ischemic stroke. Front. Aging Neurosci. 12:511562. doi: 10.3389/fnagi.2020.511562, PMID: 33192448 PMC7645221

[ref31] Ma J. Xie H. Yuan C. Shen J. Chen J. Chen Q.. (2024). The gut microbial signatures of patients with lacunar cerebral infarction. Nutr. Neurosci. 27, 620–636. doi: 10.1080/1028415x.2023.2242121, PMID: 37538045

[ref32] Oh S. E. Parikh N. S. (2022). Recent advances in the impact of infection and inflammation on stroke risk and outcomes. Curr. Neurol. Neurosci. Rep. 22, 161–170. doi: 10.1007/s11910-022-01179-6, PMID: 35235168 PMC8889053

[ref33] Peh A. O'Donnell J. A. Broughton B. R. S. Marques F. Z. (2022). Gut microbiota and their metabolites in stroke: a double-edged sword. Stroke 53, 1788–1801. doi: 10.1161/strokeaha.121.036800, PMID: 35135325

[ref34] Plovier H. Everard A. Druart C. Depommier C. Van Hul M. Geurts L.. (2017). A purified membrane protein from *Akkermansia muciniphila* or the pasteurized bacterium improves metabolism in obese and diabetic mice. Nat. Med. 23, 107–113. doi: 10.1038/nm.4236, PMID: 27892954

[ref35] Pluta R. Januszewski S. Czuczwar S. J. (2021). The role of gut microbiota in an ischemic stroke. Int. J. Mol. Sci. 22:915. doi: 10.3390/ijms22020915, PMID: 33477609 PMC7831313

[ref36] Rothwell P. M. Buchan A. Johnston S. C. (2006). Recent advances in the management of transient ischaemic attacks and minor ischaemic strokes. Lancet Neurol. 5, 323–331. doi: 10.1016/s1474-4422(06)70408-2, PMID: 16545749

[ref37] Rothwell P. M. Warlow C. P. (2005). Timing of TIAs preceding stroke: time window for prevention is very short. Neurology 64, 817–820. doi: 10.1212/01.Wnl.0000152985.32732.Ee, PMID: 15753415

[ref38] Sacco R. L. Kasner S. E. Broderick J. P. Caplan L. R. Connors J. J. Culebras A.. (2013). An updated definition of stroke for the 21st century: a statement for healthcare professionals from the American Heart Association/American Stroke Association. Stroke 44, 2064–2089. doi: 10.1161/STR.0b013e318296aeca, PMID: 23652265 PMC11078537

[ref39] Shi J. Zhao Y. Chen Q. Liao X. Chen J. Xie H.. (2023). Association analysis of gut microbiota and prognosis of patients with acute ischemic stroke in basal ganglia region. Microorganisms 11:2667. doi: 10.3390/microorganisms11112667, PMID: 38004679 PMC10673176

[ref40] Simats A. Liesz A. (2022). Systemic inflammation after stroke: implications for post-stroke comorbidities. EMBO Mol. Med. 14:e16269. doi: 10.15252/emmm.202216269, PMID: 35971650 PMC9449596

[ref41] Singh V. Roth S. Llovera G. Sadler R. Garzetti D. Stecher B.. (2016). Microbiota dysbiosis controls the neuroinflammatory response after stroke. J. Neurosci. 36, 7428–7440. doi: 10.1523/jneurosci.1114-16.2016, PMID: 27413153 PMC6705544

[ref42] Slattery C. Cotter P. D. O'Toole P. W. (2019). Analysis of health benefits conferred by *Lactobacillus* species from kefir. Nutrients 11:1252. doi: 10.3390/nu11061252, PMID: 31159409 PMC6627492

[ref43] Stöllberger C. Finsterer J. Pratter A. Kopsa W. Preiser J. Valentin A. (2003). Ischemic stroke and splenic rupture in a case of *Streptococcus bovis* endocarditis. J. Clin. Microbiol. 41, 2654–2658. doi: 10.1128/jcm.41.6.2654-2658.2003, PMID: 12791896 PMC156515

[ref44] Sun W. Huang S. Yang X. Luo Y. Liu L. Wu D. (2023). The oral microbiome of patients with ischemic stroke predicts their severity and prognosis. Front. Immunol. 14:1171898. doi: 10.3389/fimmu.2023.1171898, PMID: 37138888 PMC10150016

[ref45] Winek K. Engel O. Koduah P. Heimesaat M. M. Fischer A. Bereswill S.. (2016). Depletion of cultivatable gut microbiota by broad-Spectrum antibiotic pretreatment worsens outcome after murine stroke. Stroke 47, 1354–1363. doi: 10.1161/strokeaha.115.011800 27056982 PMC4839545

[ref46] Wu O. Cloonan L. Mocking S. J. Bouts M. J. Copen W. A. Cougo-Pinto P. T.. (2015). Role of acute lesion topography in initial ischemic stroke severity and long-term functional outcomes. Stroke 46, 2438–2444. doi: 10.1161/strokeaha.115.009643, PMID: 26199314 PMC4550548

[ref47] Xu K. Gao X. Xia G. Chen M. Zeng N. Wang S.. (2021). Rapid gut dysbiosis induced by stroke exacerbates brain infarction in turn. Gut 70, 1486–1494. doi: 10.1136/gutjnl-2020-323263, PMID: 33558272

[ref48] Xu D. J. Wang K. C. Yuan L. B. Li H. F. Xu Y. Y. Wei L. Y.. (2021). Compositional and functional alterations of gut microbiota in patients with stroke. Nutr. Metab. Cardiovasc. Dis. 31, 3434–3448. doi: 10.1016/j.numecd.2021.08.045, PMID: 34666915

[ref49] Yamashiro K. Tanaka R. Urabe T. Ueno Y. Yamashiro Y. Nomoto K.. (2017). Gut dysbiosis is associated with metabolism and systemic inflammation in patients with ischemic stroke. PLoS One 12:e0171521. doi: 10.1371/journal.pone.0171521, PMID: 28166278 PMC5293236

[ref50] Yao S. Xie H. Wang Y. Shen N. Chen Q. Zhao Y.. (2023). Predictive microbial feature analysis in patients with depression after acute ischemic stroke. Front. Aging Neurosci. 15:1116065. doi: 10.3389/fnagi.2023.1116065, PMID: 37032826 PMC10076592

[ref51] Yin J. Liao S. X. He Y. Wang S. Xia G. H. Liu F. T.. (2015). Dysbiosis of gut microbiota with reduced trimethylamine-N-oxide level in patients with large-artery atherosclerotic stroke or transient ischemic attack. J. Am. Heart Assoc. 4:e002699. doi: 10.1161/jaha.115.002699, PMID: 26597155 PMC4845212

[ref52] Yu S. Chen J. Zhao Y. Liao X. Chen Q. Xie H.. (2023). Association analysis of the gut microbiota in predicting outcomes for patients with acute ischemic stroke and H-type hypertension. Front. Neurol. 14:1275460. doi: 10.3389/fneur.2023.1275460, PMID: 37954644 PMC10639143

[ref53] Zavaglia M. Forkert N. D. Cheng B. Gerloff C. Thomalla G. Hilgetag C. C. (2015). Mapping causal functional contributions derived from the clinical assessment of brain damage after stroke. Neuroimage Clin 9, 83–94. doi: 10.1016/j.nicl.2015.07.009, PMID: 26448908 PMC4544394

[ref54] Zhang L. Liu C. Jiang Q. Yin Y. (2021). Butyrate in energy metabolism: there is still more to learn. Trends Endocrinol. Metab. 32, 159–169. doi: 10.1016/j.tem.2020.12.003, PMID: 33461886

