# Supplementary figures and images for: Specific gut microbiome signatures predict the risk of acute ischemic stroke
Source: Front Aging Neurosci. 2024 Nov 8;16:1451968. doi: 10.3389/fnagi.2024.1451968 (PMC11582031; doi:10.3389/fnagi.2024.1451968)

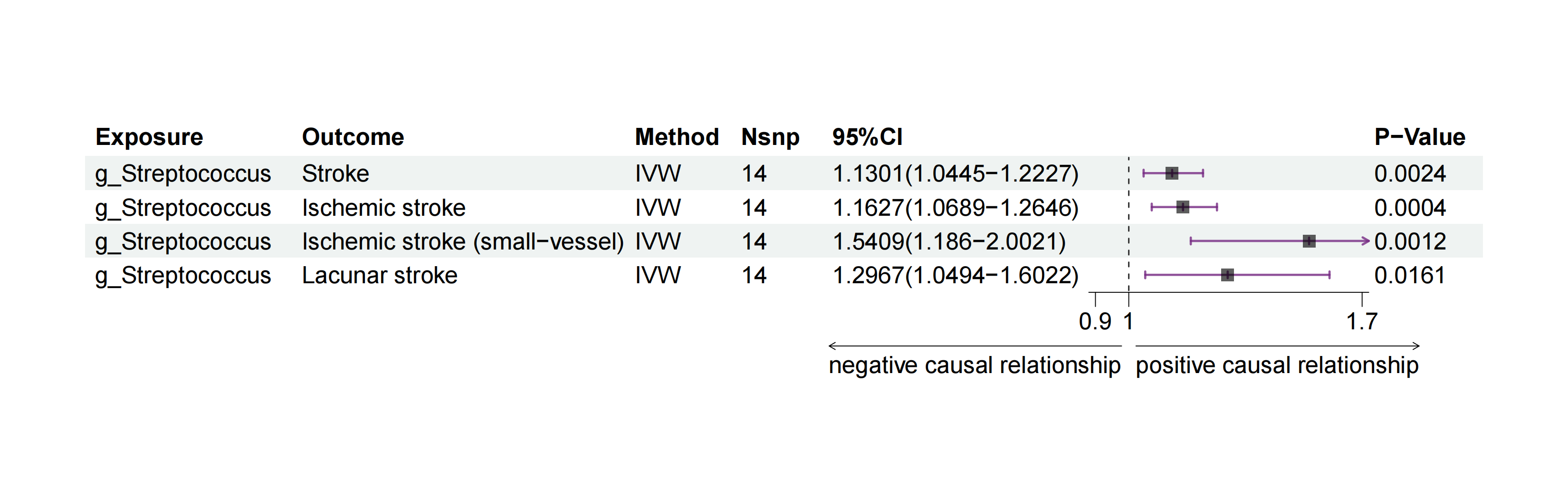

Supplement: Supplementary file 1 [file Image_1.tif]
